# Efficacy of avelumab plus axitinib for advanced renal cell carcinoma as late-line therapy: A case report

**DOI:** 10.1016/j.eucr.2022.102198

**Published:** 2022-08-24

**Authors:** Ayano Uekawa, Ryoma Kurahashi, Takanobu Motoshima, Yoji Murakami, Junji Yatsuda, Tomomi Kamba

**Affiliations:** Department of Urology, Faculty of Life Sciences, Kumamoto University, 1-1-1 Honjo, Chuo-ku, Kumamoto, 860-8556, Japan

**Keywords:** Metastatic renal cell carcinoma, Avelumab plus axitinib, Late line, IO-TKI combo, Combination therapy

## Abstract

A 46-year-old man developed a right renal tumor with multiple lung and hilar lymph node metastases. Laparoscopic radical nephrectomy was performed, and clear cell renal cell carcinoma was diagnosed 6 years earlier. Despite the use of available systemic therapeutic agents, atelectasis in the right upper lobe due to a pulmonary hilar mass and brain metastases reduced his performance, and he was becoming terminally ill. After administration of avelumab plus axitinib as 9th-line therapy, significant shrinkage of the metastases and improvement in performance status were observed. This case indicates the possibility of using avelumab plus axitinib as late-line therapy.

## 1 Introduction

In 2019, the JAVELIN Renal 101 trial (NCT02684006) demonstrated significantly longer progression-free survival (PFS) with avelumab plus axitinib than with sunitinib in patients who were previously untreated for advanced renal cell carcinoma (RCC) regardless of programmed cell death-ligand 1 (PD-L1) expression.^1^ Most of the efficacy evaluations of avelumab had been done in the first-line setting, including the JAVELIN Renal 101 trial, which was the basis for this combination therapy. However, the efficacy of avelumab plus axitinib as a late-line regimen is unknown. An advanced RCC case treated with a combination of avelumab plus axitinib as a late-line therapy, resulting in significant tumor shrinkage despite the use of multiple molecular-targeted agents, is reported.

## 2 Case report

**Fig. 1:**
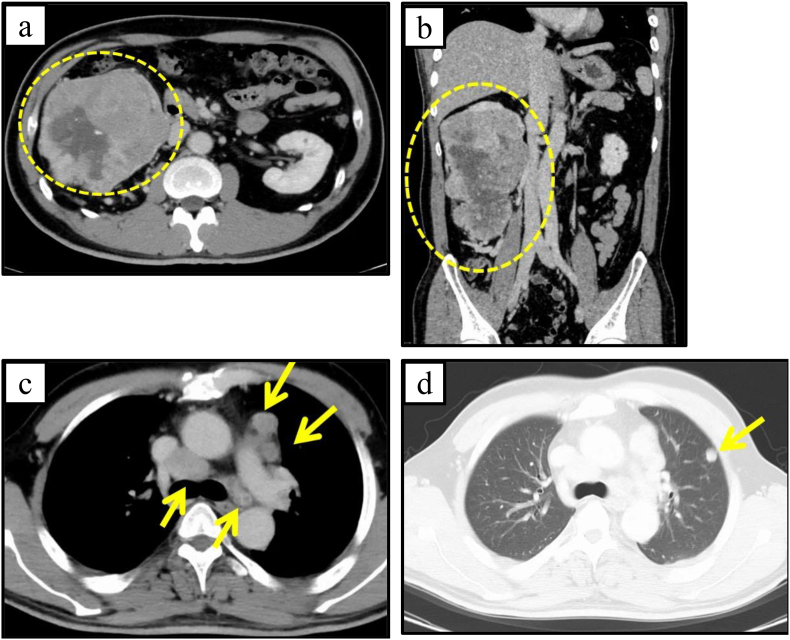
CT findings at the first visit. (a,b) Axial and coronal images show a right renal mass, strongly enhanced in the arterial phase due to hypervascularization and washed-out in the nephrogenic phase. (c) Hilar lymph nodes are enlarged. (d) Multiple lung metastases.

A 46-year-old man presented with cough and weight loss to a previous hospital 6 years earlier. Since computed tomography (CT) showed a right renal tumor, he was referred to our hospital. CT showed a right renal mass, multiple lung masses, and enlargement of hilar lymph nodes, and metastases from renal tumor were suspected (Fig. 1). For the purpose of diagnosis and tumor mass reduction, laparoscopic radical nephrectomy was performed. Pathological examination showed clear cell renal cell carcinoma (pT3aN1M1, Stage IV).

For treatment of metastases, he received 8 lines of therapies for 5 years after surgery in the following order: sunitinib, axitinib, everolimus, nivolumab, pazopanib, sorafenib, temsirolimus, and interferon-α. The best response achieved was partial response (PR) to sunitinib, nivolumab, and temsirolimus. Pazopanib was discontinued at 1 month due to severe adverse events. After treatment with interferon-α, CT showed growth of the liver, brain, and lung metastases. Swelling of the hilar lymph nodes caused a large area of right lung atelectasis, resulting in a marked decrease in Karnofsky's Performance Status (PS) to 50%, and he was terminally ill. Avelumab plus axitinib was then administered as 9th-line treatment.

**Fig. 2:**
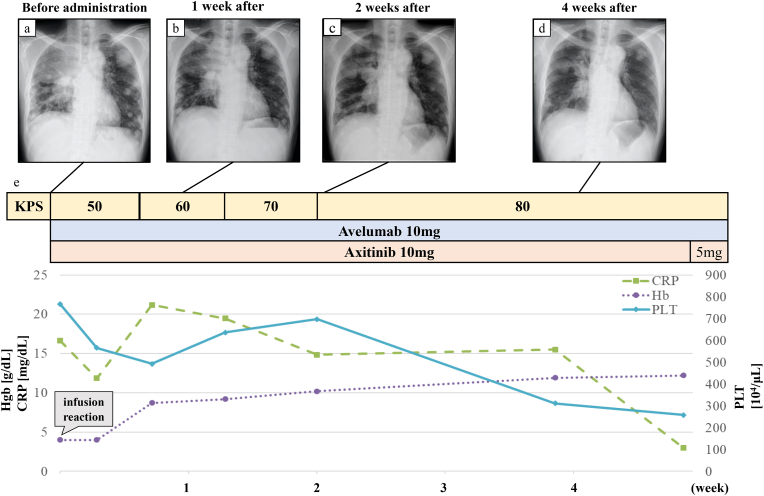
After administration of avelumab plus axitinib, the time course of hemoglobin (Hb), C-reactive protein (CRP), platelet count (PLT), and changes in X-ray findings.

After administration of avelumab plus axitinib, significant shrinkage of hilar lymph nodes was observed on X-ray, and atelectasis improved gradually. The platelet count, hemoglobin (Hb), C-reactive protein (CRP), and PS also improved gradually (Fig. 2). He could be discharged because his general condition improved.

**Fig. 3:**
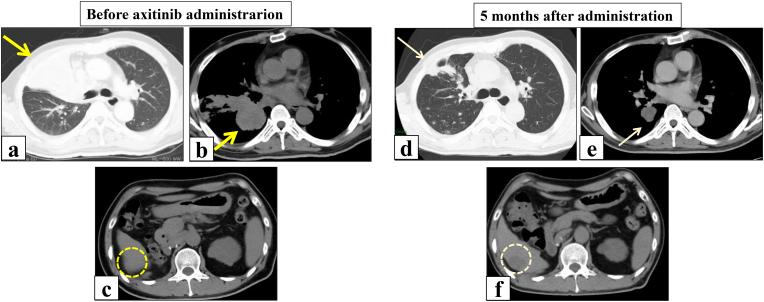
CT findings before and 5 months after administration of avelumab plus axitinib. CT shows multiple lung metastases, right pleural infiltration, pulmonary artery and vein infiltration, and right upper lobe atelectasis due to hilar lymph node enlargement (a,b). Re-expansion of the collapsed lung with shrinkage of the hilar lymph node enlargement and lung metastasis shrinkage after treatment (d,e). Liver metastasis in S6 is also seen before the treatment (c), and it has shrunk after treatment (f).

Five months after the treatment, CT showed re-expansion of the right upper collapsed lung due to shrinkage of the hilar lymph nodes and multiple lung metastases. Liver and adrenal metastases had also shrunk (Fig. 3). Unfortunately, the disease progressed, and avelumab induced immune checkpoint inhibitor (ICI)-related pneumonitis 6 months after its administration, and the patient died of the disease despite 5-month treatment with cabozantinib with a best response of SD.

## 3 Discussion

Avelumab plus axitinib showed excellent tumor suppression in the JAVELIN Renal 101 trial, indicating a new treatment strategy. Since then, several combinations of tyrosine kinase inhibitors (TKIs) and ICIs have been applied for the treatment of patients with metastatic renal cell carcinoma. The optimal selection criteria for these combination therapies do not yet exist. In the present case, avelumab plus axitinib was selected because other combination therapies had not been approved in Japan at that time.

In the present case, the patient had been treated with all molecular targeted drugs and ICIs available for renal cancer at the time of initiation of avelumab plus axitinib. He achieved PR in the early stage of the treatment, and the general condition improved significantly.

In fact, the patient received axitinib as monotherapy in an earlier line. This initial axitinib treatment demonstrated a best response of stable disease (SD), but resulted in progressive disease (PD) 8 months after administration. The reasons why rechallenge with axitinib in combination therapy was effective in this case are unclear, although there are several case reports that indicate that rechallenge of targeted agents including axitinib is feasible.^2^ One possibility is that the efficacy of this combination might have been mainly the result of avelumab, since a PD-L1 inhibitor had never been used during the course of treatment in the present case.^3^ Another possibility is that the use of nivolumab after the initial axitinib treatment might have increased sensitivity to TKIs including axitinib. Azuma et al. reported that metastatic RCC regained sensitivity to previously used axitinib after immunotherapy with nivolumab. A third re-administration with axitinib after nivolumab treatment resulted in tumor shrinkage.^4^ They proposed several mechanisms. First, ICIs and TKIs might differentially target the heterogenous cancer cell population. ICIs kill the immunotherapy-sensitive cancer cell population, permitting only the remaining TKI-sensitive cells to grow. Re-administering a TKI effectively shrinks the tumor consisting mainly of the TKI-sensitive cell population. Second, TKIs might augment ICI efficacy indirectly through several actions. ICIs resolve the anergic state of cytotoxic T lymphocytes (CTLs). These naïve CTLs could be primed by tumor antigens from the cells destroyed by the TKIs. Theses CTLs might still be suppressed by myeloid-derived suppressor cells (MDSCs). TKIs could reduce MDSCs via the inhibition of vascular endothelial growth factor (VEGF) signaling. Furthermore, it is also possible that vessel normalization and improved blood perfusion by a VEGFR-TKI could support not only drug delivery, but also CTL infiltration to tumor cells. In addition, vessel normalization could directly reduce hypoxia, resulting in the decrease in inhibitory immune signals such as PD-L1 expression on tumor cells and the conversion of the tumor microenvironment from an immunosuppressive to an immunosupportive state.^5^

## 4 Conclusion

ICI-TKI combination therapy could be effective as late-line therapy in advanced RCC even if the combination includes a re-challenged TKI. In general, ICI-TKI therapy should be used in the first line, but there is room for consideration of its use in the later line with sufficient discussion of its indication.

## Funding

This research did not receive any specific grant from funding agencies in the public, commercial, or not-for-profit sectors.

## Declaration of competing interest

The authors declare that they have no conflict of interests.
